# Neuroimaging studies of acupuncture on Alzheimer’s disease: a systematic review

**DOI:** 10.1186/s12906-023-03888-y

**Published:** 2023-02-23

**Authors:** Zihan Yin, Ziqi Wang, Yaqin Li, Jun Zhou, Zhenghong Chen, Manze Xia, Xinyue Zhang, Jiajing Wu, Ling Zhao, Fanrong Liang

**Affiliations:** 1 School of Acu-Mox and Tuina, Chengdu University of Traditional Chinese Medicine, Chengdu, China; 2 Acupuncture Clinical Research Center of Sichuan Province, Chengdu, China; 3 the Fourth People’s Hospital of Chengdu, Chengdu, China; 4 School of Nursing, Zunyi Medical University, Zunyi, China

**Keywords:** Acupuncture, Alzheimer’s disease, Systematic review, Neuroimaging

## Abstract

**Background:**

Acupuncture effectively improves cognitive function in Alzheimer’s disease (AD). Many neuroimaging studies have found significant brain alterations after acupuncture treatment of AD, but the underlying central modulation mechanism is unclear.

**Objective:**

This review aims to provide neuroimaging evidence to understand the central mechanisms of acupuncture in patients with AD.

**Methods:**

Relevant neuroimaging studies about acupuncture for AD were retrieved from eight English and Chinese medicine databases (PubMed, Embase, Cochrane Library, Web of Science, SinoMed, CNKI, WF, VIP) and other resources from inception of databases until June 1, 2022, and their methodological quality was assessed using RoB 2.0 and ROBINS - I. Brain neuroimaging information was extracted to investigate the potential neural mechanism of acupuncture for AD. Descriptive statistics were used for data analysis.

**Results:**

Thirteen neuroimaging studies involving 275 participants were included in this review, and the overall methodological quality of included studies was moderate. The approaches applied included task-state functional magnetic resonance imaging (ts-fMRI; *n* = 9 studies) and rest-state functional magnetic resonance imaging (rs-fMRI; *n* = 4 studies). All studies focused on the instant effect of acupuncture on the brains of AD participants, including the cingulate gyrus, middle frontal gyrus, and cerebellum, indicating that acupuncture may regulate the default mode, central executive, and frontoparietal networks.

**Conclusion:**

This study provides evidence of the neural mechanisms underlying the effect of acupuncture on AD involving cognitive- and motor-associated networks. However, this evidence is still in the preliminary investigation stage. Large-scale, well-designed, multimodal neuroimaging trials are still required to provide comprehensive insight into the central mechanism underlying the effect of acupuncture on AD. (Systematic review registration at PROSPERO, No. CRD42022331527).

**Supplementary Information:**

The online version contains supplementary material available at 10.1186/s12906-023-03888-y.

## Background

Alzheimer’s disease (AD), a progressive neurodegenerative disease, [1–3] initially affects cognitive function and progresses to loss of control over activities of daily living, as well as several psychological and behavioral changes [4, 5]. Dementia due to AD prevalence grows exponentially with age [6, 7]. With an annual overall incidence of 12.8–15.8 per 1000 persons, it has been estimated that the dementia population will rise to 131 million globally by 2050 [8–10]. In addition, as the current population ages, the incidence of AD increases, thus increasing the caring and nursing burden [11]. However, due to the complex pathogenesis of AD, no disease-modifying therapy is available [12, 13]; hence, AD is currently one of the most serious global healthcare and economic health concerns.

So far, there is no cure therapy for the whole AD process. Pharmacotherapy, as the current first-line treatment for AD [14], has a persistent role across all AD stages [2, 15]; however, pharmacotherapy may cause bradycardia [16], gastrointestinal disorders [17], and other adverse effects. Consequently, there is an urgent need to investigate non-pharmacological therapies for AD. Acupuncture, a commonly used non-pharmacological method in China, has long been used to treat cognitive dysfunction in China [18–21]. Numerous systematic reviews/meta-analyses [22–25] and randomized controlled trials (RCTs) [26, 27] have revealed that acupuncture can be used to treat AD. For instance, Huang et al. [23] found that acupuncture was superior to conventional medicines. Moreover, Zhou et al [25] revealed that acupuncture plus herbal medicine might have advantages over Western drugs, and our previous study [28] illustrated that acupuncture, as a monotherapy or a complementary therapy, is effective against AD.

The effect of acupuncture on AD is widely recognized; however, the underlying mechanisms have not been fully elucidated. Numerous studies have demonstrated that AD is one of the most prevalent central neurodegenerative diseases [29–31]. Brain imaging alterations have been considered the underlying pathological targets in AD [32]. Accordingly, it would be valuable to explore the central mechanisms of acupuncture in AD. With the growing number of neuroimaging studies on acupuncture for AD, multiple neuroimaging approaches and various analytical methods offer evidence of the underlying neural mechanism [33–37]. However, no systematic review has revealed the central mechanism in patients with AD. Therefore, this systematic review of neuroimaging studies aimed to evaluate the use of acupuncture in AD treatment to provide evidence for its clinical application and as a reference for future clinical research.

## Methods

### Study design

This review was registered on the PROSPERO platform (No. CRD42022331527) and improved reporting based on the Preferred Reporting Items for Systematic Review and Meta-Analysis (PRISMA) statement [38] and A Measure Tool to Assess Systematic Reviews-2 (AMSTAR-2) checklist [39].

### Inclusion and exclusion criteria

All neuroimaging clinical studies on the use of acupuncture on AD were eligible for inclusion. Conversely, we excluded case reports, comments, and studies with missing neuroimaging data. Patients with only AD with specific criteria (such as the Diagnostic and Statistical Manual of Mental Disorders (DSM), and the National Institute of Neurological and Communicative Disorders and Stroke and the Alzheimer’s Disease and Related Disorders Association (NINCDS-ADRDA) criteria), regardless of age, sex, race, or region, were included. We did not include any studies that enrolled patients diagnosed with other types of dementia or a combination of different types of dementia. Eligible interventions involved manual and electronic acupuncture, regardless of the acupoint, needling technique, treatment duration, and acupuncturist. Control methods comprised healthy control, waitlist, placebo, usual care, and conventional medicine. At least one neuroimaging approach should have been used: functional near-infrared spectroscopy (fNIRS), structural magnetic resonance imaging (sMRI), functional magnetic resonance imaging (fMRI), and positron emission tomography (PET). Since this study aimed to investigate the neuroimaging mechanism of acupuncture for AD, the outcomes were amplitude of low-frequency fluctuations (ALFF), functional connectivity (FC), and cerebral neuron alteration.

### Search strategy

Two reviewers independently searched the following sources from inception until June 1, 2022: PubMed, Embase, Cochrane Library, Web of Science (WOS), SinoMed, China National Knowledge Infrastructure (CNKI), WanFang Database, Chinese Scientific Journal Database (VIP), three clinical trial registries (WHO ICTRP (www.who.int/clinical-trials-registry-platform), ChiCTR (www.chictr.org.cn/), Clinical Trials.gov (clinicaltrials.gov)), and Grey Literature Database (www.greylit.org/). The search terms were a combination of Alzheimer’s disease, acupuncture, and neuroimaging-related terms; More detail on the search strategies is provided in Additional file 1.

### Study selection and data extraction

Duplicate studies were excluded, and potentially eligible studies were uploaded using NoteExpress V3.0. The titles, abstracts, and keywords were screened according to the inclusion criteria to identify relevant studies. Finally, the reviewers rechecked the full-text neuroimaging studies to determine their eligibility for inclusion.

Two investigators independently extracted information using a self-defined standardized extraction form that covered the identification information (first author, country, and publication date), basic information (study design, sample size, diagnostic standard, age, and sex), details about the intervention and control groups, clinical variables, and neuroimaging findings. The Standards for Reporting Interventions in Clinical Trials of Acupuncture (STRICTA) provides precise guidelines for reporting acupuncture interventions, and details on the acupuncture interventions used were collected based on these guidelines. Any disagreements were settled by consultation.

### Quality assessment

The Cochrane Handbook was applied to independently assess the risk of bias by two evaluators using the related Excel template (available from www.riskofbias.info/). The methodological quality of the RCTs was assessed based on the risk of bias 2.0 tool (RoB 2) [40]. The tool evaluates the RoB across five domains (randomization process, deviations from the intended interventions, missing data, outcome measurement, and reporting). Subsequently, an overall rating for the RoB was derived for each outcome. And The overall RoB was ranked as high, low, and some concerns. Any disagreements were settled by consultation.

The methodological quality of non-RCTs was evaluated using the risk of bias in non-randomized studies of interventions (ROBINS-I) [41]. This tool evaluates the RoB across seven factors (pre-intervention (bias due to confounding and bias in selection of participants), at-intervention (classification bias), and post-intervention (bias due to intended interventions deviations, missing data, outcome measurement, and reporting)). Subsequently, the overall RoB rating was derived for each outcome. The overall RoB was ranked as critical, serious, moderate, low, or no information.

### Statistical analysis

Because of the various analytical approach in the included studies, a descriptive analysis was used for the statistical analysis for the moment. This analysis was conducted to summarize acupuncture-induced brain alterations in patients with AD. The data are presented as counts and frequencies.

## Results

### Study description

#### Literature search

**Fig. 1:**
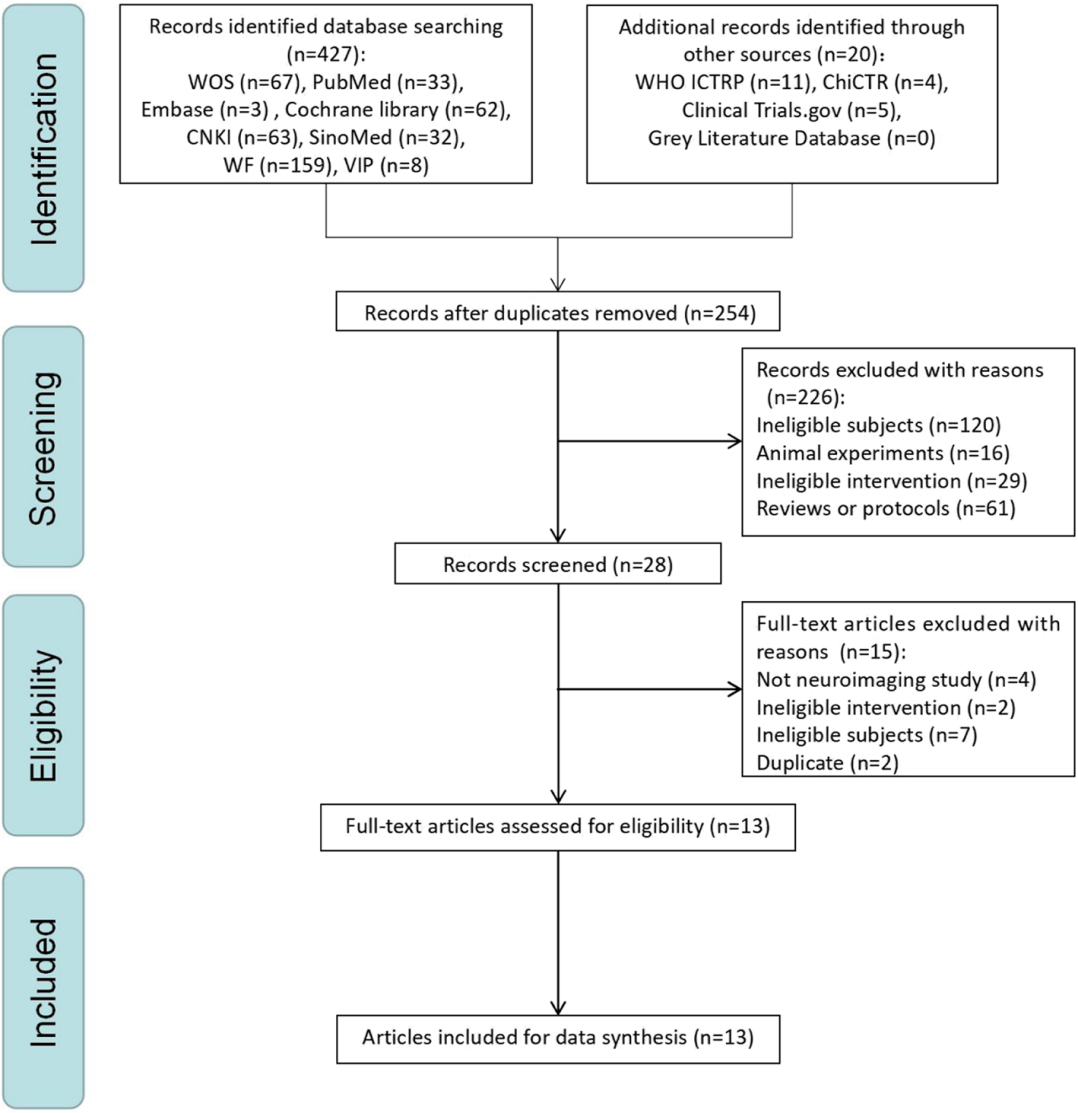
The PRISMA flow chart of selection process

From the literature search, we identified 447 potentially eligible trials. After removing any duplicates, 254 studies were included. Next, the titles, abstracts, and keywords of the remaining publications were reviewed against the exclusion criteria, which left 28 potential candidate studies. Finally, after reading the full-text, 15 trials were excluded (four due to not being neuroimaging studies, two with ineligible treatment, seven with ineligible subjects, and two with duplicate content), leaving 13 trials [34–36, 42–51]. The PRISMA flow chart for this review is depicted in Fig. 1, and the reasons why the 15 full-text studies were excluded are listed in Additional file 2.

#### Study characteristics

Table 1 lists the details and main characteristics of the 13 studies included in the current review. The published neuroimaging data were from 2005 to 2021. All of the included studies were performed in China.

**Table 1:** Main characteristics of included neuroimaging studies

Study	Country	Study design	N (A/B/C)	Diagnostic criteria	Age (Year)	Gender (M/F)	Severity	Treatment courses	(A)	(B)	(C)	Imaging modality	Scan T	Clinical variables	Outcomes
									Intervention Group	Control Group I	Control Group II				
Ji 2021 [42]	China	Non-RCT	24 (12/12)	①②	A: 67.58 ± 9.05 B: 64.83 ± 6.94	A: 7/5 B: 7/5	Mild and moderate	3 minutes	Acupuncture	HC	/	rs-fMRI	3.0 T	MoCA, AVLT, CDR	FC
Shan 2018 [43]	China	RCT	35(14/7/14)	①②	A: 66.93 ± 8.91 B: 71.29 ± 4.75 C: 66.07 ± 5.78	A: 4/10 B: 5/2 C: 6/8	/	3 minutes	Acupuncture	Sham acupuncture	HC	ts-fMRI	3.0 T	/	Brain activation
Zheng 2018 [34]	China	Non-RCT	28 (14/14)	①②	A: 66.92 ± 8.91 B: 66.07 ± 5.78	A: 6/8 B: 6/8	Mild and moderate	3 minutes	Acupuncture	HC	/	rs-fMRI	3.0 T	MoCA, MMSE, AVLT, CDR	ALFF, FC, and correlation with neuropsychological measures
Wang 2014 [36]	China	Non-RCT	28 (14/14)	①②	A: 66.92 ± 8.91 B: 66.07 ± 5.78	A: 4/10 B: 6/8	Mild and moderate	3 minutes	Acupuncture	HC	/	rs-fMRI	3.0 T	/	FC
Liang 2014 [35]	China	Non-RCT	20 (9/11)	①②	A: 65.11 ± 9.84 B: 66.45 ± 5.55	A: 3/6 B: 3/8	Mild and moderate	3 minutes	Acupuncture	HC	/	rs-fMRI	3.0 T	MoCA, MMSE	FC, and correlation with neuropsychological measures
Wang 2012 [44]	China	Non-RCT	22 (8/14)	①②	A: 66.92 ± 8.91 B: 66.07 ± 5.78	A: 3/5 B: 6/8	Mild and moderate	3 minutes	Acupuncture	HC	/	ts-fMRI	3.0 T	MMSE, AVLT, CDR	Brain activation
Zhou 2008 [45]	China	Non-RCT	26	②	71.6	16/10	Mild and moderate	6 minutes	Acupuncture	/	/	ts-fMRI	1.5 T	/	Brain activation
Fu 2006 [46]	China	Non-RCT	20 (10/10)	②	/	/	Mild and moderate	12 minutes	Acupuncture	HC	/	ts-fMRI	2.0 T	/	Brain activation
Yan 2005 [47]	China	Non-RCT	24 (11/13)	②	A: 65.8 ± 9.7 B: 62.0 ± 6.1	A: 4/7 B: 5/8	8 cases of mild and 3 cases of moderate	/	Acupuncture	HC	/	ts-fMRI	1.5 T	/	Brain activation
Wang 2005 [48]	China	Non-RCT	24 (11/13)	①②	A: 65.5 ± 9.5 B: 62.2 ± 6.1	A: 4/7 B: 7/6	4 cases of mild, 5 cases of moderate, and 2 cases of severe	3 minutes	Acupuncture	HC	/	ts-fMRI	1.5 T	/	Brain activation
Fu 2005a [49]	China	Non-RCT	6	②	/	/	Mild and moderate	3 minutes	Acupuncture	/	/	ts-fMRI	1.5 T	/	Brain activation
Fu 2005b [50]	China	Non-RCT	6	②	65-80	4/2	/	3 minutes	Acupuncture	/	/	ts-fMRI	1.5 T	/	Brain activation
Fu 2005c [51]	China	Non-RCT	12 (6/6)	②	65-80	A: 2/4 B: 3/3	/	3 minutes	Acupuncture	HC	/	ts-fMRI	1.5 T	/	Brain activation

①: Diagnostic and Statistical Manual of Mental Disorders; ②: National Institute of Neurological and Communicative Disorders and Stroke and the Alzheimer’s Disease and Related Disorders Association criteria; *RCT* Randomized controlled trial, *HC* Healthy control, *ts-fMRI* Task-state functional magnetic resonance imaging, *rs-fMRI* Task-state functional magnetic resonance imaging, *ALFF* Amplitude of low-frequency fluctuation, *FC* Functional connectivity, *MMSE* The Mini-Mental State Examination, *MoCA* Montreal Cognitive Assessment Scale, *CDR* Clinical Dementia Rating, *AVLT* Auditory Verbal Learning Test

#### Study design

There was one RCT [34–36, 42–51] and 12 non-RCTs (self-control studies) [34–36, 42, 44–51] included. Nine studies [34–36, 42, 44–51] explored acupuncture-induced brain activation, four studies [34–36, 42] investigated acupuncture-induced brain functional networks, and one study [34] evaluated acupuncture-induced brain neuronal activity.

#### Participants

A total of 154 patients with AD and 121 healthy subjects were included, and all neuroimaging studies applied the NINCDS-ADRDA criteria [52]. Seven studies [34–36, 42–44, 48] applied DSM [9] and NINCDS-ADRDA criteria. Nine studies [34–36, 42, 44, 46–49] compared patients with AD to healthy subjects. Three studies [45, 50, 51] only enrolled patients with AD, while one trial [45, 50, 51] used a three-group design containing acupuncture, sham acupuncture, and healthy controls. The sample sizes ranged from six to 35, and 275 participants were included in the study. Only one study [43] had a sample size of more than 30. The common matching sample size ratio of AD/health controls was 1:1. The subjects with AD ranged in age from 62 to 80 years, and 12 studies [43] reported AD patient sex (62 males and 76 females). Nine neuroimaging studies [34–36, 42, 44–47, 50] included patients with mild and moderate AD, and one study [48] enrolled patients with mild to severe AD.

#### Acupuncture

The acupuncture details of the neuroimaging studies were collected and are displayed in Table 2 on account of the STRICTA [53]. The rationale for acupuncture was reported in all of the neuroimaging studies. The subjects had between one to four needle insertions per session, and LR 3 (eight studies, 61.54%) and LI 4 (eight studies, 61.54%) were the commonly applied acupoints. The mean value of the insertion depth was 14.31, and all acupuncture depths were not more than 25 mm. Seven studies described the responses sought. Manual acupuncture was employed in eight studies. Electronic acupuncture was used in five studies, and the electronic acupuncture apparatus used was G6805C. Typically, the needles used in both acupuncture methods were 0.30 mm in diameter and 25 mm long. The studies involved a one treatment session of 3 min. Only two studies provided details of the acupuncturists, and nine reported details of the comparator interventions.

**Table 2:** Details of acupuncture methods according to Standards for Reporting Interventions in Clinical Trials of Acupuncture (STRICTA)

Study	Acupuncture rationale			Details of needling							Treatment regimen		Other components		Practitioner	Comparator interventions	
	1a	1b	1c	2a	2b	2c	2d	2e	2f	2 g	3a	3b	4a	4b	5	6a	6b
Ji 2021 [42]	TCM	Y	NA	4	LR 3 (bilateral), LI 4 (bilateral)	10-15 mm	Deqi	Manual	3 minutes	Diameter and length: 0.30 mm & 25 mm Needle brand: Cloud & Dragon	1	Frequency: once Duration: 3 minutes	NA	NR	NR	Y	Y
Shan 2018 [43]	TCM	Y	NA	4	LR 3 (bilateral), LI 4 (bilateral)	NR	NR	Manual	3 minutes	NR	1	Frequency: once Duration: 3 minutes	NA	NR	NR	Y	NR
Zheng 2018 [34]	TCM	Y	NA	4	LR 3 (bilateral), LI 4 (bilateral)	NR	NR	Manual	3 minutes	Diameter and length: 0.30 mm & 25 mm Needle brand: NR	1	Frequency: once Duration: 3 minutes	NA	NR	NR	Y	Y
Wang 2014 [36]	TCM	Y	NA	4	LR 3 (bilateral), LI 4 (bilateral)	NR	NR	Manual	3 minutes	Diameter and length: 0.30 mm & 25 mm Needle brand: NR	1	Frequency: once Duration: 3 minutes	NA	NR	NR	Y	Y
Liang 2014 [35]	TCM	Y	NA	4	LR 3 (bilateral), LI 4 (bilateral)	NR	NR	Manual	3 minutes	Diameter and length: 0.30 mm & 25 mm Needle brand: NR	1	Frequency: once Duration: 3 minutes	NA	NR	NR	Y	Y
Wang 2012 [44]	TCM	Y	NA	4	LR 3 (bilateral), LI 4 (bilateral)	NR	NR	Manual	3 minutes	Diameter and length: 0.30 mm & 25 mm Needle brand: NR	1	Frequency: once Duration: 3 minutes	NA	NR	NR	Y	Y
Zhou 2008 [45]	TCM	Y	NA	4	HT 7, ST 36, ST40, KI 3 (unilateral, left)	5-15 mm	Deqi	Electronic	6 minutes	Diameter and length: 0.30 mm & 50 mm Needle brand: Great Wall Electroacupuncture apparatus: G6805C	1	Frequency: once Duration: 6 minutes	NR	NR	NR	NA	NA
Fu 2006 [46]	TCM	Y	NA	1	KI 3 (unilateral, left)	12 mm	Deqi	Electronic	12 minutes	Diameter and length: 0.30 mm & 25 mm Needle brand: NR Electroacupuncture apparatus: G6805C	1	Frequency: twice Duration: 12 minutes	NA	NR	NR	Y	Y
Yan 2005 [47]	TCM	Y	NA	4	LR 3 (bilateral), LI 4 (bilateral)	15 mm	Deqi	Manual	NR	Diameter and length: 0.30 mm & 25 mm Needle brand: NR	1	Frequency: once Duration: NR	NA	NR	Y	Y	Y
Wang 2005 [48]	TCM	Y	NA	4	LR 3 (bilateral), LI 4 (bilateral)	25 mm	Deqi	Manual	3 minutes	Diameter and length: 0.30 mm & 25 mm Needle brand: NR	1	Frequency: once Duration: 3 minutes	NA	NR	Y	Y	Y
Fu 2005a [49]	TCM	Y	NA	1	HT 7 (unilateral, right)	10 mm	NR	Electronic	3 minutes	Diameter and length: 0.30 mm & 25 mm Needle brand: NR Electroacupuncture apparatus: G6805C	1	Frequency: once Duration: 3 minutes	NA	NR	NR	NA	NA
Fu 2005b [49]	TCM	Y	NA	1	PC 6 (unilateral, right)	10 mm	Deqi	Electronic	3 minutes	Diameter and length: 0.30 mm & 25 mm Needle brand: NR Electroacupuncture apparatus: G6805C	1	Frequency: once Duration: 3 minutes	NA	NR	NR	NA	NA
Fu 2005c [50]	TCM	Y	NA	1	PC 6 (unilateral, right)	20 mm	Deqi	Electronic	3 minutes	Diameter and length: 0.30 mm & 25 mm Needle brand: NR Electroacupuncture apparatus: G6805C	1	Frequency: once Duration: 3 minutes	NA	NR	NR	Y	Y

1a: Style of acupuncture; 1b: Reasoning for treatment provided; 1c: Extent to which treatment was varied; 2a: Number of needle insertions per subject per session; 2b: Names of points used; 2c: Depth of insertion; 2d: Response sought; 2e: Needle stimulation; 2f: Needle retention time; 2 g: Needle type; 3a: Number of treatment sessions; 3b: Frequency and duration of treatment sessions; 4a: Details of other interventions administered to the acupuncture group; 4b: Setting and context of treatment; 5: Description of participating acupuncturists; 6a: Rationale for the control or comparator; 6b: Precise description of the control or comparator, *TCM* Traditional Chinese medicine, *NA* Not application, *NR* Not recorded, *Y* Yes, *&* And

#### Comparison

The 13 studies that referred to the three comparison models were as follows: acupuncture versus healthy volunteers (*n* = nine studies), the self-control model (pre- vs. post-treatment, *n* = three studies), and acupuncture versus sham acupuncture (*n* = one study).

#### Clinical variables

Four cognitive assessment approaches were used to evaluate AD: the Montreal Cognitive Assessment Scale (MoCA; three studies), Mini-Mental State Examination (MMSE; three studies), Clinical Dementia Rating (CDR; three studies), and auditory verbal learning test (AVLT; three studies).

#### Quality assessment

The one RCT included was found to have a moderate RoB using the RoB 2 approach. In the section on the “randomization process” and “selection of the reported results”, the study had some concerns due to unclear random sequence generation and lacking protocol/registration. Of the 12 non-RCTs, the ROBINS-I revealed low methodological quality in five studies and high quality in seven. Four studies had serious risks in the confounding section due to insufficient baseline confounding details (such as cognitive function and education level), whereas seven had a low risk. Regarding missing data, one study had a serious risk due to insufficient details on large sample losses in the follow-up period, while others had a low risk. Notably, all studies had a low RoB in the other sections. The results of the RoB evaluations are presented in Additional files 3 and 4.

### Neuroimaging findings of acupuncture for AD

#### Imaging condition and analysis

Functional magnetic resonance imaging (fMRI) was used to measure functional changes induced by acupuncture, including task-state fMRI (ts-fMRI) and rest-state fMRI (rs-fMRI). ts-fMRI was applied to explore brain functional activation, whereas rs-fMRI was applied to explore brain functional networks using the FC method, and brain neuronal activity using the ALFF.

#### Acupuncture-related brain activities in fMRI

All studies focused on the instant effect of acupuncture. Rest and task states were the two main study designs used for fMRI. The neuroimaging results after acupuncture are presented in Additional file 5.

Based on the ts-fMRI studies, nine neuroimaging studies [34–36, 42] reported that the activated brain areas after acupuncture concerned the processing of cognitive function, including the memory regions (e.g., the hippocampus, inferior frontal gyrus, superior frontal gyrus, and superior temporal gyrus), auditory speech area (e.g., the superior temporal gyrus, transverse temporal gyrus, and inferior parietal lobule), language function region (e.g., the middle frontal gyrus, middle temporal gyrus, and cerebellum), spatial attention region (e.g., the superior parietal lobule), affective-emotional processing areas of cognitive function (e.g., the insula, cingulate cortex, and thalamus), motor function region (e.g., the precentral gyrus), and sensory function region (e.g., the postcentral gyrus).

Regarding the four rs-fMRI studies [34–36, 42], one study [34] reported that increased ALFF after acupuncture in the brain area occurred in the postcentral gyrus, and decreased ALFF in the brain area occurred in the inferior frontal gyrus, hippocampus, and cingulate cortex. In addition, using FC analysis, Ji et al. [42] found that the right middle frontal gyrus of the right frontal-parietal network decreased significantly after acupuncture. Conversely, two studies [34, 36] reported increased FC between the hippocampus and middle frontal gyrus/precentral gyrus. Furthermore, one study [35] found an increased connection between cognition-related brain areas, such as the inferior parietal lobule, middle temporal gyrus, and posterior cingulate cortex, with decreased connectivity between the cingulate gyrus and precuneus.

#### The relationship between the neuroimaging results and clinical outcomes

Furthermore, there was a significant positive correlation between the FC strength of the right middle temporal gyrus and the changed MMSE and MoCA scores [35]. Meanwhile, Zheng et al. [35] found positive correlations between the ALFF of the subgenual cingulate cortex and MMSE and MoCA scores, with negative correlations between the AVLT scores and ALFF of the hippocampus and the right inferior temporal gyrus or FC of the right hippocampus and the left precentral gyrus.

#### Top ten acupuncture-induced altered brain areas

As demonstrated in Table 3, the top ten acupuncture-related brain alterations in subjects with AD were in the cingulate gyrus (eight studies, 61.54%), middle frontal gyrus (seven studies, 53.85%), cerebellum (seven studies, 53.85%), superior temporal gyrus (six studies, 46.15%), inferior parietal lobule (six studies, 46.15%), superior frontal gyrus (five studies, 38.46%), inferior frontal gyrus (five studies, 38.46%), middle temporal gyrus (five studies, 38.46%), postcentral gyrus (four studies, 30.77%), superior parietal lobule (three studies, 23.08%), precentral gyrus (three studies, 23.08%), and hippocampus (three studies, 23.08%).

**Table 3:** Top 10 brain areas of acupuncture-related alterations of included neuroimaging studies

Brain area	Counts	Percentage
Cingulate gyrus	8	61.54%
Middle frontal gyrus	7	53.85%
Cerebellum	7	53.85%
Superior temporal gyrus	6	46.15%
Inferior parietal lobule	6	46.15%
Superior frontal gyrus	5	38.46%
Inferior frontal gyrus	5	38.46%
Middle temporal gyrus	5	38.46%
Postcentral gyrus	4	30.77%
Superior parietal lobule	3	23.08%
Precentral gyrus	3	23.08%
Hippocampus	3	23.08%

#### Potential acupuncture-induced altered brain pathways

**Fig. 2:**
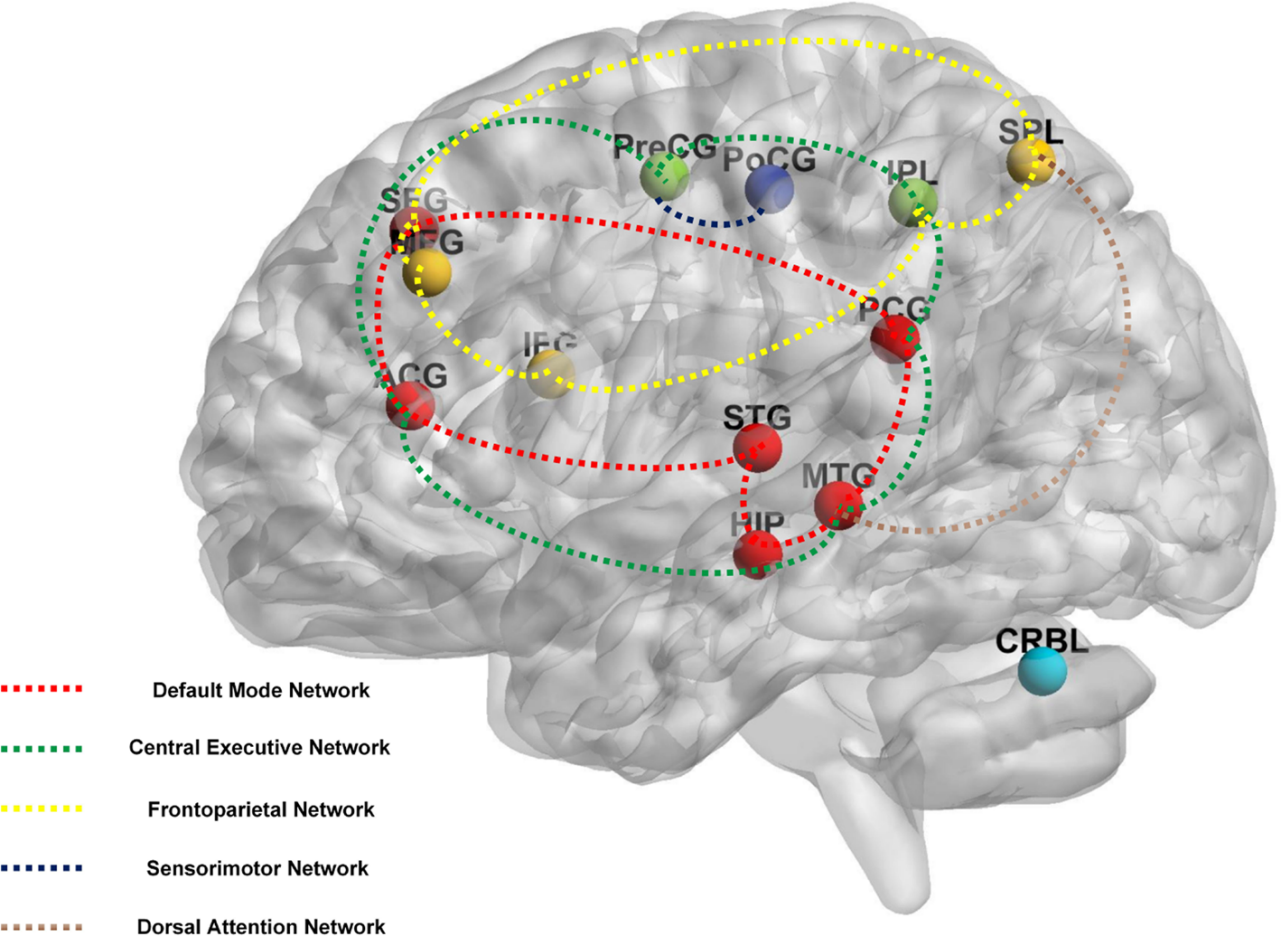
The main reported neuroimaging findings by acupuncture. Note. ACG: anterior cingulate and paracingulate gyri; CRBL: cerebellum; HIP: hippocampus; IFG: inferior frontal gyrus; IPL: inferior parietal lobule; MFG: middle frontal gyrus; MTG: middle temporal gyrus; PCG: posterior cingulate gyrus; PoCG: postcentral gyrus; PreCG: precentral gyrus; SFG: superior frontal gyrus; SPL: superior parietal lobule; STG: superior temporal gyrus

The main brain areas could be roughly classified into five pathways (Fig. 2, Additional file 5): the default mode network (DMN; for example, the cingulate gyrus, superior temporal gyrus, superior frontal gyrus, middle temporal gyrus, and hippocampus), central executive network (CEN; for example, the cingulate gyrus, inferior parietal lobule, middle temporal gyrus, and precentral gyrus), frontoparietal network (FPN; for example, the middle frontal gyrus, inferior parietal lobule, superior frontal gyrus, inferior frontal gyrus, superior parietal lobule), dorsal attention network (DAN; for example, the middle temporal gyrus, superior parietal lobule), and the sensorimotor network (SMN; for example, the postcentral gyrus, and precentral gyrus).

## Discussion

### Study characteristics of the acupuncture neuroimaging studies on AD

The included studies aimed to explore whether acupuncture could cause brain activation, alternation of the brain network, and brain neuronal activity. Nine of the 13 neuroimaging studies explored whether acupuncture could cause brain activation. However, there is no consensus regarding these alterations. In addition, four studies investigated whether acupuncture could affect brain functional networks, and one study evaluated acupuncture-induced brain neuronal activity. However, only two studies determined the correlation between acupuncture effects and functional network/neuronal activity.

Based on previous study [54], the sample size may affect the treatment effect evaluation. All included studies had a sample size of less than 30, which may have affected the stability and replicability of the findings; thus, future studies should be conducted with larger sample sizes to confirm the findings of the current review. Additionally, there were more female than male with AD. According to previous studies [55–57], sex is a vital feature influencing physiopathological mechanisms and therapies for patients with AD. Nevertheless, no study explored on the sex-disaggregated neural mechanism of acupuncture in AD. Therefore, sex-disaggregated neuroimaging trials are supposed to carry out.

In terms of the acupuncture details, of the included 13 neuroimaging studies, LR 3 (Taichong) and LI 4 (Hegu) LR 3 and LI 4 were the most frequently used acupoints. In traditional Chinese medicine theory, LI 4 and LR 3 are named the Siguan [58]. Siguan points are recommended for future neuroimaging studies on acupuncture interventions in AD. In addition, eight studies adopted manual acupuncture, but only two recorded the details of the acupuncturists. Researchers should pay more attention to acupuncturists [59, 60]. Moreover, the cerebral response is associated with the degree of sensation in neuroimaging; consequently, the response sought should be carefully documented. Finally, based on the STRICTA, several factors may have affected the study findings; therefore, future studies should apply standardized acupuncture procedures based on the STRICTA.

Only three comparison models, acupuncture versus healthy volunteers, self-controlled before and after acupuncture, and acupuncture versus sham acupuncture, were adopted in the included studies. The acupuncture versus healthy volunteer model explores the distinct cerebral activities between patients with AD and healthy people before and after acupuncture, the self-control comparison investigates the alterations in brain areas affected by acupuncture, and acupuncture versus sham acupuncture evaluates the specific cerebral alterations between acupuncture and placebo. However, according to the STRICTA criteria, these models are insufficient for exploring the numerous effects of acupuncture in AD. For instance, according to the STRICTA, the depth, response sought, acupuncture stimulation, practitioners, and other factors affecting acupuncture efficacy require further research.

Regarding the outcome measurements, the cognitive outcome measures applied in the included studies were MMSE, MoCA, CDR, and AVLT, which have been commonly applied to detect cognitive impairment. MMSE and CDR are suggested for evaluating dementia, MoCA is recommended for evaluating mild cognitive impairment (MCI) [61], and AVLT is recommended to measure episodic memory function in AD [62]. However, none of these scales comprehensively assess the cognitive function of patients with AD. The Alzheimer’s Disease Assessment Scale-Cognitive Subscale (ADAS-Cog), as a gold standard, was developed to assess the level of cognitive dysfunction in AD [63, 64]. Accordingly, it is recommended that ADAS-Cog be used to assess the cognitive function of patients with AD in future studies.

### Current methodology of the included studies

Seven of the 13 neuroimaging studies were designated as low risk; five studies were ranked as serious risk; and others were ranked as some concerns about the quality of the methodology used. The one RCT included had some concerns due to issues in the randomization process and selection of the reported results. Of the 12 non-RCTs included, the risk of bias in five studies was serious due to the potential bias of confounding and measurement of the outcomes. Therefore, future studies should strictly conform to the Cochrane Handbook. For instance, according to the Cochrane Handbook, future RCTs should pay more attention to implementing the randomization process and selecting the reported results. Future non-RCTs should provide sufficient details regarding confounding and missing data. Also, due to the unsatisfactory methodological quality, it is suggested that more high quality neuroimaging studies should be conducted to improve the quality and confirm the findings of the current review. In addition, RCTs are considered the gold standard for evaluating the efficacy of interventions; thus, more RCTs are required to confirm that acupuncture protocols employed in neuroimaging studies are effective in AD treatment.

### The neuroimaging findings of acupuncture for AD

All of the 13 studies included concentrated solely on brain functional alterations. The neuroimaging method used in the included studies was fMRI. fMRI indirectly measures brain alterations to study the nervous system via hemodynamic and neurovascular coupling [65]. According to previous studies [66–69], AD is a multidimensional central nervous system disease that affects brain structure and function. Although fMRI is a powerful approach for brain activity analysis, it is not comprehensive enough for neural processing. Numerous neuroimaging techniques have been used to assess neural processing in AD. For instance, diffusion tensor imaging (DTI) is an advanced MRI technique that has been used to provide qualitative and quantitative white matter microarchitecture information of AD [70]. Furthermore, electroencephalography (EEG), the measurement of the brain’s electric fields, has been used in diagnosing, assessing, and monitoring medical treatment in patients with AD [71]. PET, a tool used to quantify physiological processes, has proven useful for diagnosing and predicting AD [72]. It is well known that these approaches have their own characteristics, so integrating multiple approaches allows for a more comprehensive assessment of the effects of acupuncture on AD. Thus, integrating multiple approaches (such as fMRI with DTI, fMRI with EEG, and fMRI with PET) can be a comprehensive way to study acupuncture in AD. Multimodal neuroimaging methods are urgently needed to provide an opportunity to understand the comprehensive neural mechanisms of acupuncture for AD and guide clinical treatment options for patients with AD.

In this review, all of the included studies also explored the instant acupuncture effect, demonstrating that the top ten brain regions affected were the cingulate gyrus, middle frontal gyrus, cerebellum, superior temporal gyrus, inferior parietal lobule, superior frontal gyrus, inferior frontal gyrus, middle temporal gyrus, postcentral gyrus, superior parietal lobule, precentral gyrus, and hippocampus. These brain areas are associated with AD. Accumulating evidence [73, 74] suggests that the cingulate gyrus is a neurodegenerative biomarker for understanding the neural mechanisms of AD. In addition, numerous studies [75–77] have reported increased FC in the middle frontal gyrus and other brain regions in AD. Moreover, previous reports [78–81] have illustrated that the cerebellum contributes to cognitive and neuropsychiatric deficits in AD. Previous studies have revealed that the cortical thickness of the superior temporal gyrus and inferior parietal lobule changes during AD progression [82, 83]. In addition, the superior frontal gyrus and inferior frontal gyrus were positively associated with cognitive function [77, 84]. Moreover, word fluency and naming were correlated with the cortical thickness of the middle temporal gyrus [85]. Valera-Bermejo also found that the postcentral and precentral gyri volumes were correlated with episodic and semantic memory [86]. Accumulating research [87–89] has illustrated a change in FC in the superior parietal lobule and other brain regions in AD. Numerous studies have demonstrated that the hippocampus, one of the first structures affected by AD, was regarded as a sensitive neurodegenerative neuroimaging biomarker [90–92]. Meanwhile, previous neuroimaging studies [93–98] illustrated that adjusting these brain regions is a crucial neural mechanism of acupuncture treatment in cognitive impairment diseases. Since acupuncture can promote neuroplasticity and repair these damaged brain areas [99, 100], the changes in these brain regions are due to an interaction between acupuncture and AD pathology. In addition, the top ten brain areas of most included studies were emerged naturally with data-driven approaches, and only one region (hippocampus) was specifically analysed in a study [36]. Therefore, these brain areas are genuinely changing with acupuncture in AD compared to other regions. Based on the findings, future acupuncture studies should pay more attention to these brain regions.

The included neuroimaging studies of acupuncture for AD showed that instant acupuncture might adjust the brain networks. Significant pathways related to acupuncture for AD in the included studies are displayed. The results showed that the essential regions of DMN, CEN, FPN, DAN, and SMN are included in the brain regions that undergo alterations due to acupuncture for AD. These brain networks are correlated with cognitive and motor function. DMN plays an important role in cognitive function and internally directed thoughts. Moreover, it is closely connected with AD due to its association with AD atrophy modes and tau sedimentation [101–103]. CEN is a significant network obsessed with cognitive control and episodic memory [104, 105]. DAN is involved in the externally oriented actions and perceptions [106–108]. Numerous studies have demonstrated that altered DMN, CEN, and DAN are prominent biomarkers of AD [109]. FPN was shown to play an outstanding role in executive and language functions [110, 111]. Many studies [110–113] have illustrated that abnormal FC and compensation in the FPN might coexist in AD. SMN was characterized by a hypoactivation phase in patients with AD [114], while the altered FC of the sensorimotor cortical network was associated with a phenotype conversion from MCI to AD [115]. Previous studies [116, 117] have demonstrated disrupted large-scale resting-state FCs in the above networks in patients with AD. Meanwhile, multiple neuroimaging studies [118–122] have suggested that regulating the alterations of these networks is a vital mechanism of acupuncture treatment. Therefore, cognitive-related and motor-associated brain areas are involved in the acupuncture mechanism of AD, implying that acupuncture may modulate these associated networks.

### Strengths and weaknesses

To our knowledge, previous systematic reviews have focused on the efficacy and safety of acupuncture for AD. This is the first systematic review to explore the acupuncture mechanism for AD. Moreover, this review may provide specific insights into the neurocentral mechanism of acupuncture in AD subjects by summarizing the findings of recent clinical neuroimaging studies. In addition, the review was registered in PROSPERO and followed the PRISMA and AMSTAR-2 statement to improve the reporting and methodological quality. Nonetheless, this review had several limitations. First, the studies included used multiple analytical imaging approaches; therefore, quantitative meta-analyses were impossible. Furthermore, while 13 studies were included, only one was an RCT, and the others were non-RCTs, indicating low evidence. Due to the small sample size of the included studies, the findings are potentially biased. Moreover, the included studies only focused on the effects of instant acupuncture. Nevertheless, while the instant effect of acupuncture is still important to consider, the constant acupuncture effect must be explored. Finally, acupuncture has not only been used in clinical practice but is usually combined with other therapies for AD; accordingly, studies that investigate the mechanism of acupuncture combined with other therapies for AD should be conducted in the future.

## Conclusion

This review indicates that acupuncture for AD involves brain regions in cognitive- and motor-associated networks, especially in the cingulate gyrus, middle frontal gyrus, and cerebellum. However, these findings remain in the preliminary exploration stage. Larger, well-designed, long-term trials with multimodal neuroimaging techniques need to be conducted to confirm the neuroimaging findings.

## Supplementary Information

**Additional file 1.** Search strategies of each database.

**Additional file 2.** Full-text articles excluded with reasons.

**Additional file 3.** Methodological quality assessments of randomised studies of the effects of interventions using RoB 2.

**Additional file 4.** Methodological quality assessments of non-randomised studies of the effects of interventions using ROBINS-I.

**Additional file 5.** Neuroimaging results after acupuncture.

## Data Availability

All data generated or analysed during this study are included in this article.
