# Supplementary material for: Neuroimaging studies of acupuncture on Alzheimer’s disease: a systematic review
Source: BMC Complement Med Ther. 2023 Feb 23;23:63. doi: 10.1186/s12906-023-03888-y (PMC9948384; doi:10.1186/s12906-023-03888-y)
Supplement: Supplementary file 1 — Additional file 1. Search strategies of each database. [file 12906_2023_3888_MOESM1_ESM.docx]

**Appendix 1. Search strategies of each database.**

**PubMed**

#1 "Alzheimer Disease"[Mesh]

#2 "Alzheimer Disease" [Title/Abstract] OR "Alzheimer's Disease" [Title/Abstract] OR "Alzheimer Dementia" [Title/Abstract] OR "Alzheimer*" [Title/Abstract] OR "Alzheimer Type Dementia" [Title/Abstract] OR "Senile Dementia" [Title/Abstract]

#3 #1 OR #2

#4 "Acupuncture therapy"[Mesh]

#5 "acupuncture therapy"[Title/Abstract] OR "acupuncture"[Title/Abstract] OR "acupuncture-moxibustion"[Title/Abstract] OR "meridian*"[Title/Abstract] OR "acupoint*"[Title/Abstract] OR "warm needling"[Title/Abstract] OR "warm acupuncture"[Title/Abstract] OR "acupuncture plus moxibustion"[Title/Abstract] OR "electronic acupuncture"[Title/Abstract] OR "electro-acupuncture"[Title/Abstract] OR "electroacupuncture"[Title/Abstract] OR "fire acupuncture"[Title/Abstract] OR "auricular needle"[Title/Abstract] OR "scalp needle"[Title/Abstract] OR "abdominal needle"[Title/Abstract] OR "wrist ankle needle"[Title/Abstract] OR "triple puncture"[Title/Abstract] OR "dry needle"[Title/Abstract] OR "needle"[Title/Abstract] OR "body acupuncture"[Title/Abstract] OR "manual-acupuncture"[Title/Abstract]

#6 #4 OR #5

#7 "neuroimaging"[MeSH] OR "magnetic resonance imaging"[Mesh] OR "Positron-Emission Tomography"[Mesh] OR "magnetic resonance imaging"[Mesh]

#8 "neuroimaging"[Title/Abstract] AND "magnetic resonance imaging"[Title/Abstract] OR "Positron-Emission Tomography"[Title/Abstract] OR "functional magnetic resonance imaging"[Title/Abstract] OR "fMRI"[Title/Abstract] OR "functional image"[Title/Abstract] OR "Diffusion Tensor Imaging"[Title/Abstract] OR "DTI"[Title/Abstract] OR "structural magnetic resonance imaging"[Title/Abstract] OR "sMRI"[Title/Abstract] OR "functional near-infrared spectroscopy"[Title/Abstract] OR "electroencephalography"[Title/Abstract] OR "magnetic resonance spectroscopy"[Title/Abstract] OR "magnetoencephalography"[Title/Abstract]

#9 #7 OR #8

#10 #3 AND #6 AND #9

**EMBASE**

#1 'alzheimer disease'/exp/mj

#2 'alzheimer disease':ti,ab,kw OR 'alzheimers disease':ti,ab,kw OR 'alzheimer dementia':ti,ab,kw OR alzheimer:ti,ab,kw OR 'alzheimer type dementia':ti,ab,kw OR 'senile dementia':ti,ab,kw

#3 #1 OR #2

#4 'acupuncture therapy'/exp/mj

#5 'acupuncture therapy':ti,ab,kw OR acupuncture:ti,ab,kw OR 'acupuncture moxibustion':ti,ab,kw OR meridian*:ti,ab,kw OR acupoint*:ti,ab,kw OR 'warm needling':ti,ab,kw OR 'warm acupuncture':ti,ab,kw OR 'acupuncture plus moxibustion':ti,ab,kw OR 'electronic acupuncture':ti,ab,kw OR 'electro acupuncture':ti,ab,kw OR electroacupuncture:ti,ab,kw OR 'fire acupuncture':ti,ab,kw OR 'auricular needle':ti,ab,kw OR 'scalp needle':ti,ab,kw OR 'abdominal needle':ti,ab,kw OR 'wrist ankle needle':ti,ab,kw OR 'triple puncture':ti,ab,kw OR 'dry needle':ti,ab,kw OR needle:ti,ab,kw OR 'body acupuncture':ti,ab,kw OR 'manual acupuncture':ti,ab,kw

#6 #4 OR #5

#7 'neuroimaging'/exp OR 'magnetic resonance imaging'/exp OR 'Positron-Emission Tomography'/exp OR 'magnetic resonance imaging'/exp

#8 neuroimaging:ti,ab,kw OR magnetic resonance imaging:ti,ab,kw OR Positron-Emission Tomography:ti,ab,kw OR functional magnetic resonance imaging:ti,ab,kw OR fMRI:ti,ab,kw OR functional image:ti,ab,kw OR Diffusion Tensor Imaging:ti,ab,kw OR DTI:ti,ab,kw OR structural magnetic resonance imaging:ti,ab,kw OR sMRI:ti,ab,kw OR functional near-infrared spectroscopy:ti,ab,kw OR electroencephalography:ti,ab,kw OR magnetic resonance spectroscopy:ti,ab,kw OR magnetoencephalography:ti,ab,kw

#9 #7 OR #8

#10 #3 AND #6 AND #9

**Web of science**

TS=(‘Alzheimer Disease’ OR ‘Alzheimer's Disease’ OR ‘Alzheimer Dementia’ OR ‘Alzheimer*’ OR ‘Alzheimer Type Dementia’ OR ‘Senile Dementia’) AND (‘acupuncture*’ OR ‘acupuncture-moxibustion’ OR ‘meridian*’ OR ‘acupoint*’ OR ‘warm needling’ OR ‘warm acupuncture’ OR ‘acupuncture plus moxibustion’ ) AND (‘neuroimaging’ OR ‘magnetic resonance imaging’ OR ‘Positron-Emission Tomography’ OR ‘functional magnetic resonance imaging’ OR ‘fMRI’ OR ‘functional image’ OR ‘Diffusion Tensor Imaging’ OR ‘DTI’ OR ‘structural magnetic resonance imaging’ OR ‘sMRI’ OR ‘functional near-infrared spectroscopy’ OR ‘electroencephalography’ OR ‘magnetic resonance spectroscopy’ OR ‘magnetoencephalography’)

**Cochrane Library**

#1 Mesh descriptor: [Alzheimer Disease]explode all trees

#2 alzheimer disease:ti,ab,kw OR alzheimers disease:ti,ab,kw OR alzheimer dementia:ti,ab,kw OR alzheimer:ti,ab,kw OR alzheimer type dementia:ti,ab,kw OR senile dementia:ti,ab,kw OR ad:ti,ab,kw OR atd:ti,ab,kw

#3 #1 OR #2

#4 Mesh descriptor: [acupuncture therapy] explode all trees;

#5 acupuncture therapy:ti,ab,kw OR acupuncture:ti,ab,kw OR acupuncture moxibustion:ti,ab,kw OR meridian*:ti,ab,kw OR acupoint*:ti,ab,kw OR warm needling:ti,ab,kw OR warm acupuncture:ti,ab,kw OR acupuncture plus moxibustion:ti,ab,kw OR electronic acupuncture:ti,ab,kw OR electro acupuncture:ti,ab,kw OR electroacupuncture:ti,ab,kw OR fire acupuncture:ti,ab,kw OR auricular needle:ti,ab,kw OR scalp needle:ti,ab,kw OR abdominal needle:ti,ab,kw OR wrist ankle needle:ti,ab,kw OR triple puncture:ti,ab,kw OR dry needle:ti,ab,kw OR needle:ti,ab,kw OR body acupuncture:ti,ab,kw OR manual acupuncture:ti,ab,kw

#6 #4 OR #5

#7 Mesh descriptor: [neuroimaging] explode all trees

#8 Mesh descriptor: [magnetic resonance imaging] explode all trees

#9 Mesh descriptor: [Positron-Emission Tomography] explode all trees

#10 Mesh descriptor: [magnetic resonance imaging] explode all trees

#11 neuroimaging:ti,ab,kw OR magnetic resonance imaging:ti,ab,kw OR Positron-Emission Tomography:ti,ab,kw OR functional magnetic resonance imaging:ti,ab,kw OR fMRI:ti,ab,kw OR functional image:ti,ab,kw OR Diffusion Tensor Imaging:ti,ab,kw OR DTI:ti,ab,kw OR structural magnetic resonance imaging:ti,ab,kw OR sMRI:ti,ab,kw OR functional near-infrared spectroscopy:ti,ab,kw OR electroencephalography:ti,ab,kw OR magnetic resonance spectroscopy:ti,ab,kw

#12 #7 OR #8 OR #9 OR #10 OR #11

#13 #3 AND #6 AND #12

**CNKI Strategy in English**

(TKA=(‘acupuncture’+‘acupuncture moxibustion’+‘body acupuncture’+‘manual acupuncture’+‘electronic acupuncture’+‘warm acupuncture’+‘scalp needle’+‘scalp acupuncture’+‘auricular needle’+‘acupoint’+‘fire acupuncture’+‘abdominal needle’+‘float needle’+‘three-edged needle’+‘nine needle’+‘meridian’+‘transcutaneous electrical stimulation’+‘eye acupuncture’+‘tongue acupuncture’+‘wrist ankle needle’+‘blade needle’+‘acupotomy’+‘micropuncture’+‘dry needle’) OR SU=(‘Acupuncture therapy' + 'Acupuncture' + 'acupuncture-moxibustion therapy’)) AND (TKA=(‘Alzheimer Disease’+‘Senile Dementia’+‘Alzheimer's Disease’+‘Senile Type Dementia’) OR SU=(‘cognitive impairment’+‘Alzheimer Disease’)) AND (TKA=(‘neuroimaging’+‘brain imaging’+‘magnetic resonance imaging’+‘functional magnetic resonance imaging’+‘structural magnetic resonance imaging’+‘Diffusion Tensor Imaging’+‘PET’+‘functional near-infrared spectroscopy’+‘electroencephalography’+‘magnetoencephalography’) OR SU=(‘neuroimaging’+‘brain imaging’))

**WF Strategy in English**

(Title or Keywords:(Alzheimer Disease OR Alzheimer's Disease OR Senile Dementia OR Senile Type Dementia) OR Subject:(cognitive impairment OR Alzheimer Disease)) and (Title or Keywords:(acupuncture OR acupuncture moxibustion OR body acupuncture OR manual acupuncture OR electronic acupuncture OR warm acupuncture OR scalp needle OR scalp acupuncture OR auricular needle OR acupoint OR fire acupuncture OR abdominal needle OR float needle OR three-edged needle OR nine needle OR meridian OR transcutaneous electrical stimulation OR eye acupuncture OR tongue acupuncture OR wrist ankle needle OR blade needle OR acupotomy OR micropuncture OR dry needle) OR Subject:(Acupuncture therapy OR Acupuncture OR acupuncture-moxibustion therapy)) and (Title or Keywords:(neuroimaging OR brain imaging OR magnetic resonance imaging OR functional magnetic resonance imaging OR structural magnetic resonance imaging OR Diffusion Tensor Imaging OR PET OR functional near-infrared spectroscopy OR electroencephalography OR magnetoencephalography) OR Subject:(neuroimaging OR brain imaging))

**Chongqing VIP** **Strategy in English**

M=(Alzheimer Disease OR Alzheimer's Disease OR Senile Dementia OR Senile Type Dementia) and M=(acupuncture OR acupuncture moxibustion OR body acupuncture OR manual acupuncture OR electronic acupuncture OR warm acupuncture OR scalp needle OR scalp acupuncture OR auricular needle OR acupoint OR fire acupuncture OR abdominal needle OR float needle OR three-edged needle OR nine needle OR meridian OR transcutaneous electrical stimulation OR eye acupuncture OR tongue acupuncture OR wrist ankle needle OR blade needle OR acupotomy OR micropuncture OR dry needle) and R=(neuroimaging OR brain imaging OR magnetic resonance imaging OR functional magnetic resonance imaging OR structural magnetic resonance imaging OR Diffusion Tensor Imaging OR PET OR functional near-infrared spectroscopy OR electroencephalography OR magnetoencephalography)

**SinoMed Strategy in English**

1 "Alzheimer Disease" [weighted:expansion]

2 "Alzheimer Disease"[Common field: Intelligence] OR "Alzheimer's Disease"[Common field: Intelligence] OR "Senile Dementia"[Common field: Intelligence] OR "Senile Type Dementia"[Common field: Intelligence] OR "Dementia"[Common field: Intelligence]

3 1 OR 2

4 "acupuncture therapy"[weighted:expansion] OR "acupuncture"[weighted:expansion] OR "acupuncture moxibustion therapy"[weighted:expansion]

5 "acupuncture"[Common field: Intelligence] OR "acupuncture moxibustion"[Common field: Intelligence] OR "body acupuncture"[Common field: Intelligence] OR "manual acupuncture"[Common field: Intelligence] OR "electronic acupuncture"[Common field: Intelligence] OR "warm acupuncture"[Common field: Intelligence] OR "scalp needle"[Common field: Intelligence] OR "scalp acupuncture"[Common field: Intelligence] OR "auricular needle"[Common field: Intelligence] OR "acupoint"[Common field: Intelligence] OR "fire acupuncture"[Common field: Intelligence] OR "abdominal needle"[Common field: Intelligence] OR "float needle"[Common field: Intelligence] OR "meridian"[Common field: Intelligence] OR "transcutaneous electrical stimulation"[Common field: Intelligence] OR "dry needle"[Common field: Intelligence]

6 4 OR 5

7 "neuroimaging" [weighted:expansion] OR "brain imaging"[weighted:expansion]

8 "neuroimaging"[Common field: Intelligence] OR "brain imaging"[Common field: Intelligence] OR "magnetic resonance imaging"[Common field: Intelligence] OR "functional magnetic resonance imaging"[Common field: Intelligence] OR "structural magnetic resonance imaging"[Common field: Intelligence] OR "Diffusion Tensor Imaging"[Common field: Intelligence] OR "PET"[Common field: Intelligence] OR "functional near-infrared spectroscopy"[Common field: Intelligence] OR "electroencephalography"[Common field: Intelligence] OR "magnetoencephalography"[Common field: Intelligence]

9 7 OR 8

10 3 AND 6 AND 9
