# Supplementary material for: Neuroimaging studies of acupuncture on Alzheimer’s disease: a systematic review
Source: BMC Complement Med Ther. 2023 Feb 23;23:63. doi: 10.1186/s12906-023-03888-y (PMC9948384; doi:10.1186/s12906-023-03888-y)
Supplement: Supplementary file 2 — Additional file 2. Full-text articles excluded with reasons. [file 12906_2023_3888_MOESM2_ESM.docx]

**Appendix 2. Full-text articles excluded with reasons.**

| Full-text articles excluded | Reasons |
| --- | --- |
| Chen 2020 [1] | Not neuroimaging study |
| Xu 2021 [2] | Not neuroimaging study |
| Wang 2014 [3] | Not neuroimaging study |
| Ye 2012 [4] | Not neuroimaging study |
| Cao 2020 [5] | Ineligible subjects |
| Li 2020 [6] | Ineligible subjects |
| He 2018 [7] | Ineligible subjects |
| Xu 2013 [8] | Ineligible subjects |
| Liu 2012 [9] | Ineligible subjects |
| Liu 2009 [10] | Ineligible subjects |
| Xu 2004 [11] | Ineligible subjects |
| Guo 2017 [12] | Ineligible intervention |
| Zhou 2008 [13] | Ineligible intervention |
| Shan 2016 [14] | Duplicate content |
| Han 2007 [15] | Duplicate content |

**References**

1. Chen Y, Li Z, Wu J: **Clinical Research of Acupoint Intradermic Injection of Xueshuantong in the Treatment of AD**. *J Clin Acu-Mox,* 2020, **36**(02):50-55.
2. Xu L: **Efficacy of acupuncture combined with Donepezil hydrochloride tablets in the treatment of Alzheimer's disease and its influence on inflammatory factors and electroencephalogram**. *China's Naturopathy* 2021, **29**(15):67-69.
3. Wang Y, Qin W, Yu C: **Clinical observation on effect of cranial suture acupuncture combined with donepezil hydrochloride tablets for Alzheimer’s disease**. *World Journal of Acupuncture-Moxibustion* 2014(2):19-24.
4. Ye J, Liu G: **Therapeutic effect of acupuncture combined with Bushen Yiqi Huoxue prescription on senile dementia**. *Journal of Sichuan of Traditional Chinese Medicine* 2012, **30**(08):138-139.
5. Cao J, Huang Y, Meshberg N, Hodges SA, Kong J: **Neuroimaging-Based Scalp Acupuncture Locations for Dementia**. *J CLIN MED* 2020, **9**(8).
6. Li H, Wang Z, Yu H, Pang R, Ni H, Li CR, Li K: **The Long-Term Effects of Acupuncture on Hippocampal Functional Connectivity in aMCI with Hippocampal Atrophy: A Randomized Longitudinal fMRI Study**. *NEURAL PLAST* 2020, **2020**.
7. He J, Zhao C, Liu W, Huang J, Liang S, Chen L, Tao J: **Neurochemical changes in the hippocampus and prefrontal cortex associated with electroacupuncture for learning and memory impairment**. *INT J MOL MED* 2018, **41**(2):709-716.
8. Xu M: **To explore the bidirectional adjustment mechanism of acupuncture at Taixi point based on fMRI**. *Doctor.*: *Guangzhou University of Chinese Medicine;* 2013.
9. Liu B, Chen J, Wang J, Liu X, Duan X, Shang X, Long Y, Chen Z, Li X, Huang Y *et al*: **Altered Small-World Efficiency of Brain Functional Networks in Acupuncture at ST36: A Functional MRI Study**. *PLOS ONE* 2012, **7**(6).
10. Liu P, Zhang Y, Zhou G, Yuan K, Qin W, Zhuo L, Liang J, Chen P, Dai J, Liu Y *et al*: **Partial correlation investigation on the default mode network involved in acupuncture: An fMRI study**. *NEUROSCI LETT* 2009, **462**(3):183-187.
11. Xu J, Wang F, Shan B, Chen Yan, Wang H, Mao X, Wang Q: **PET and fMRI to evaluate the results of acupuncture treatment of the cognition of Alzheimer’s disease.** *Chinese Imaging Journal of Integrated Traditional and Western Medicine* 2004, **2**(2):85-87.
12. Guo Z, Liu X, Cao Y, Hou H, Chen X, Chen Y, Huang F, Chen W: **Common H-1-MRS Characteristics in Patients With Alzheimer's Disease and Vascular Dementia Diagnosed With Kidney Essence Deficiency Syndrome: A Preliminary Study**. *ALTERN THER HEALTH M* 2017, **23**(3):12-18.
13. Zhou YL, Han HY, Jia JP: **Correlation analysis on changes between cognitive ability and brain fMRI after acupoint thread embedding in Alzheimer's disease patients**. 2008, **28**(8):689-693.
14. Shan Y, Bian Y, Wang Z, Zhao Z, Qi Z, Qiu B, Lu J, Li K: **Functional magnetic resonance study on acupoint specificity of acupuncture at Siguan point in the treatment of Alzheimer's disease.** In: *Chinese Congress of Radiology 2016:*2016*; Suzhou*; 2016: 1.
15. Han H: **The Functionary Magnetic Resonance Image Detects the Brain Activation of the Acupoint Embedding Catgut treating the Alzheimer's Disease**. *Master.*: Henan University of Traditional Chinese Medicine; 2007.
