# Supplementary material for: Neuroimaging studies of acupuncture on Alzheimer’s disease: a systematic review
Source: BMC Complement Med Ther. 2023 Feb 23;23:63. doi: 10.1186/s12906-023-03888-y (PMC9948384; doi:10.1186/s12906-023-03888-y)
Supplement: Supplementary file 3 — Additional file 3. Methodological quality assessments of randomised studies of the effects of interventions using RoB 2. [file 12906_2023_3888_MOESM3_ESM.docx]

**Appendix 3. Methodological quality assessments of randomised studies of the effects of interventions using RoB 2.**

| Study | Randomisation process | Deviations from intended interventions | Missing outcomes data | Measurement of the outcome | Selection of the reported results | Overall |
| --- | --- | --- | --- | --- | --- | --- |
| Shan 2018 | Some concerns① | Low | Low | Low | Some concerns② | Some concerns |

**Notes:** ① unclear random sequence generation; ②insufficient details of protocol/registration.
