# Supplementary material for: Neuroimaging studies of acupuncture on Alzheimer’s disease: a systematic review
Source: BMC Complement Med Ther. 2023 Feb 23;23:63. doi: 10.1186/s12906-023-03888-y (PMC9948384; doi:10.1186/s12906-023-03888-y)
Supplement: Supplementary file 4 — Additional file 4. Methodological quality assessments of non-randomised studies of the effects of interventions using ROBINS-I. [file 12906_2023_3888_MOESM4_ESM.docx]

**Appendix 4.** **Methodological quality assessments of non-randomised studies of the effects of interventions using ROBINS-I.**

| Study | Bias due to confounding | Bias in selection of participants into study | Bias in classification of interventions | Bias due to deviations from intended interventions | Bias due to missing data | Bias in measurement of outcomes | Bias in selection of the reported result | Overall |
| --- | --- | --- | --- | --- | --- | --- | --- | --- |
| Ji 2021 | Low | Low | Low | Low | Low | Low | Low | Low |
| Zheng 2018 | Low | Low | Low | Low | Low | Low | Low | Low |
| Wang 2014 | Low | Low | Low | Low | Low | Low | Low | Low |
| Liang 2014 | Low | Low | Low | Low | Serious② | Low | Low | Serious |
| Wang 2012 | Low | Low | Low | Low | Low | Low | Low | Low |
| Zhou 2008 | Low | Low | Low | Low | Low | Low | Low | Low |
| Fu 2006 | Serious① | Low | Low | Low | Low | Low | Low | Serious |
| Yan 2005 | Low | Low | Low | Low | Low | Low | Low | Low |
| Wang 2005 | Low | Low | Low | Low | Low | Low | Low | Low |
| Fu 2005a | Serious① | Low | Low | Low | Low | Low | Low | Serious |
| Fu 2005b | Serious① | Low | Low | Low | Low | Low | Low | Serious |
| Fu 2005c | Serious① | Low | Low | Low | Low | Low | Low | Serious |

**Notes:** ① insufficient details of baseline confounding (such as cognitive function, education level); ② Large losses to follow up from the acupuncture and control groups. Reasons for loss to follow up from both groups not stated.
