# Supplementary material for: Neuroimaging studies of acupuncture on Alzheimer’s disease: a systematic review
Source: BMC Complement Med Ther. 2023 Feb 23;23:63. doi: 10.1186/s12906-023-03888-y (PMC9948384; doi:10.1186/s12906-023-03888-y)
Supplement: Supplementary file 5 — Additional file 5. Neuroimaging results after acupuncture. [file 12906_2023_3888_MOESM5_ESM.docx]

**Appendix 5.** **Neuroimaging results after acupuncture.**

| **Study** | **Neuroimaging technologies** | **Neuroimaging results** |
| --- | --- | --- |
| Ji 2021 | rs-fMRI (FC) | AD patients before needling vs. HC: *increased* *FC* of the left superior frontal gyrus, the left middle frontal gyrus, left precuneus, the left inferior frontal gyrus, and right middle frontal gyrus located in the left frontal parietal network; the right angular gyrus and right middle frontal gyrus in the right frontal parietal network; the left inferior parietal lobule, bilateralpostcentral gyrus, and right middle frontal gyrus in the sensorimotor network; *decreased* *FC* of right middle occipital gyrus of visual network.  AG after needling vs. AG before needling: *decreased* *FC* of right middle frontal gyrus of right frontal parietal network.  AG after needling vs. HC after needling: *increased* *FC* of left middle frontal gyrus of left frontal parietal network and the right lingual gyrus of visual network. |
| Zheng 2018 | rs-fMRI (ALFF, FC) | AD patients before needling vs. HC: *increased* *ALFF* of subgenual cingulate cortex, right middle cingulate cortex, right inferior frontal gyrus, right hippocampus and right inferior temporal gyrus, and *decreased* *ALFF* in the right superior frontal gyrus and the left postcentral gyrus.  AG after needling vs. AG before needling: *increased ALFF* of left postcentral gyrus; *decreased ALFF* of right inferior frontal gyrus, right hippocampus and middle cingulate cortex; *increased* *FC* between the right hippocampus and the left precentral gyrus. |
| Wang 2014 | rs-fMRI (FC) | AD patients before needling vs. HC: *decreased FC* of the right medial prefrontal cortex with left hippocampus, right hippocampus with right superior temporal gyrus and the inferior temporal gyrus.  AG after needling vs. AG before needling: *increased* *FC* of right middle frontal gyrus with the left hippocampus. |
| Liang 2014 | rs-fMRI (FC) | AD patients before needling vs. HC: *increased* *FC* of left cingulate gyrus, and *decreased* *FC* of the bilateral posterior cingulate cortex, bilateral precuneus, right inferior parietal lobule.  AG after needling vs. AG before needling: *increased* *FC* of right inferior parietal lobule, right middle temporal gyrus, and left posterior cingulate cortex; *decreased* *FC* of bilateral cingulate gyrus and left precuneus. |
| Shan 2018 | ts-fMRI (CNA) | AG during needling vs. AG before needling: *positive activation areas* of the bilateral cerebellum, right inferior frontal gyrus, right middle temporal gyrus, left pallidum, left rolandic operculum, left superior parietal gyrus, and left supramarginal gyrus; *negative activation areas* of the right cuneus, right pallidum, right inferior occipital gyrus, left rectus, left cerebellum, and left putamen. |
| Wang 2012 | ts-fMRI (CNA) | AD patients before needling vs. HC: *negative activation areas* of the left temporal lobe and left middle frontal gyrus.  AG during needling vs. AG before needling: *positive activation areas* of the right cerebellum posterior lobe, bilateral frontal lobe, right inferior parietal lobule, right middle occipital lobe; *negative activation areas* of the right superior temporal gyrus, right middle temporal gyrus, bilateral middle frontal gyrus and left brain stem.  AG after needling vs. AG before needling: *positive activation areas of* the right cerebellum posterior lobe, temporal lobe (left inferior temporal gyrus, right middle temporal gyrus), frontal lobe (bilateral superior frontal gyrus, left inferior frontal gyrus, right middle frontal gyrus and bilateral precentral gyrus), occipital lobe (right middle occipital lobe), parietal lobe (bilateral supramarginal gyrus, right superior parietal lobule); *negative activation areas* of the left cerebellum posterior lobe, bilateral parahippocampus, right middle frontal gyrus, left lingual gyrus, right cingulate gyrus, left lentiform nucleus and right midbrain. |
| Zhou 2008 | ts-fMRI (CNA) | AG after needling vs. AG before needling: *activation areas of* the bilateral hippocampus, bilateral insula, bilateral middle temporal gyrus, bilateral superior temporal gyrus, left transverse temporal gyrus, left superior parietal lobule, right inferior parietal lobule, right postcentral gyrus, left middle occipital gyrus, left thalamus. |
| Fu 2006 | ts-fMRI (CNA) | AG after needling vs. AG before needling: *activation areas* of bilateral superior frontal gyrus, left inferior parietal lobule, bilateral claustrum, left globus pallidus, bilateral cingulate gyrus, bilateral lingual gyrus, right middle frontal gyrus, right superior temporal gyrus, right inferior parietal lobule, right subthalamic nucleus. |
| Yan 2005 | ts-fMRI (CNA) | AG after needling vs. AG before needling: *positive activation areas* of bilateral occipital and temporal regions, and bilateral cerebellum, the left middle frontal gyrus, left superior frontal gyrus, left inferior parietal lobule, right precentral gyrus, right superior temporal gyrus, right inferior frontal gyrus, right inferior temporal gyrus, right middle occipital gyrus and right insula. *Negative activation areas* of bilateral inferior parietal lobule, bilateral occipital lobe, bilateral precentral gyrus, left occipital lobe, left anterior cingulate gyrus, right posterior central gyrus, right superior frontal gyrus, right temporal pole, right parahippocampal gyrus. |
| Wang 2005 | ts-fMRI (CNA) | AG after needling/duration vs. AG before needling: *activation areas* of bilateral prefrontal lobe cortex, cerebellum, occipital lobe, superior parietal lobule. |
| Fu 2005a | ts-fMRI (CNA) | AG after needling vs. AG before needling: *activation areas* of left middle frontal gyrus, left superior temporal gyrus, left Heschl's gyri, left cingulate gyrus, left anterior and posterior central gyrus, thalamus, right middle frontal gyrus, right superior temporal gyrus. |
| Fu 2005b | ts-fMRI (CNA) | AG after needling vs. AG before needling: *activation areas* bilateral middle temporal gyrus, bilateral superior frontal gyrus, bilateral inferior frontal gyrus, bilateral cerebellum, bilateral cingulate gyrus, right superior temporal gyrus. |
| Fu 2005c | ts-fMRI (CNA) | AG after needling vs. AG before needling: *positive activation areas* of the frontal lobe, temporal lobe, cingulate gyrus, cerebellum. |

Notes: AD: Alzheimer’s disease; HC: healthy control; AG, acupuncture group; rs-fMRI: rest state - functional magnetic resonance imaging; ts-fMRI: task state - functional magnetic resonance imaging; ALFF: amplitude of low frequency fluctuations; FC: functional connectivity; CNA: cerebral neurons alteration; MMSE: the Mini-Mental State Examination; MoCA: Montreal Cognitive Assessment Scale.
